# Silver-Mixed Port Reduces Venous Access Port Related Infection Rate Compared to Non-Silver-mixed Port: A Single-center Retrospective Analysis

**DOI:** 10.1007/s00270-023-03583-y

**Published:** 2023-10-30

**Authors:** Takayuki Suzuki, Kenkichi Michimoto, Jun Hasumi, Shunsuke Kisaki, Yasuaki Hasegawa, Ayako Fujimori, Lynn Yoshimatsu, Hirokazu Ashida, Hiroya Ojiri

**Affiliations:** 1 Department of Radiology, The Jikei University School of Medicine, The Jikei University of Second Building 14F, 3-25-8, Nishi-Shinbashi, Minato-ku, Tokyo 105-0003 Japan; 2 Department of Radiology, The Jikei University Katsushika Medical Center, 6-41-2, Aoto, Katsushika-ku, Tokyo 125-8506 Japan; 3 Department of Radiology, The Jikei University Daisan Hospital, 4-11-1, Izumihonchou, Komae-shi, Tokyo 201-8601 Japan

**Keywords:** Port infection, Silver-mixed, Gram-negative rod, Antibacterial effect

## Abstract

**Purpose:**

Totally implantable venous access ports (TIVAPs) are increasingly used as safe and convenient central venous access devices. However, several TIVAP-related complications occur, with port/catheter infection being most common. Silver-mixed ports have recently been introduced in anticipation of reducing TIVAP infection. This study aimed to investigate the efficacy of this device in reducing port infection by examining groups with and without silver-mixed devices.

**Materials and Methods:**

From April 2017 to July 2022, silver-mixed ports (S group) and non-silver-mixed port group (NS group) were reviewed at our institution. The incidence of TIVAP-related infections, patient characteristics, and bacteriological data were evaluated. Univariate and multivariate analyses were used to evaluate risk factors for TIVAP-related infection.

**Results:**

A total of 607 patients (S group, n = 203; NS group, n = 404) were enrolled. The rates of TIVAP-related infection were 3.0% (n = 6) and 7.7% (n = 31) in the S and NS groups, respectively. The incidence of total infection per 1000 catheter-days were 0.114 and 0.214 the S and NS groups, respectively. In the entire group, the rates of infection were 6.1% (n = 37) and the incidence of total infection per 1000 catheter-days was 0.187. Univariate and multivariate analyses revealed a significantly lower TIVAP-related infection rate in S group than NS group (*p* = 0.0216, odds ratio = 2.88 confidence interval: 1.17–7.08). No gram-negative rods were detected in the S group as port infection.

**Conclusion:**

Silver-mixed port may be feasible in preventing port infection.

Level of evidence.

Level 3, Local non-random sample.

## Introduction

Totally implantable venous access ports (TIVAPs) are increasingly used as a safe and convenient central venous access device for chemotherapy administration and intravenous nutrition. Various TIVAP-related complications, including pneumothorax and arterial puncture during TIVAP implantation, fibrin formation, intraluminal thrombus, catheter rupture, port tank inversion, skin ulcers at the port tank, and port/catheter infections after TIVAP implantation, have been reported [1, 2]. Among these complications, port/catheter infection is the most common.

Silver, utilized as a strong and broad-spectrum antimicrobial agent with low toxicity to humans, possesses the ability to release ions from its surface, bind bacterial cell structures, and impair outer cell layers, which result in loss of cell contents and structural abnormalities [3, 4]. Several silver-coated medical devices, such as urethral catheters coated with silver alloy in short-term placement [5] and silver-coated implants for total hip arthroplasty, have demonstrated clinically feasible anti-infectious activity [6].

Ports and catheters made of silicone mixed with silver-based inorganic antimicrobial agents (silver-mixed port; Argyle Fukuroi™ Microneedle Port Silver Type; Cardinal Health, Dublin, OH) have recently been introduced in anticipation of reducing TIVAP infection after implantation. The silver is mixed entirely in the device. The catheter tip contains a flap. This single-center retrospective study aimed to evaluate the clinical efficacy of silver-mixed venous access ports for TIVAP-related infections by examining groups with and without silver-mixed devices.

## Materials and Methods

### Study Design and Population

This single-center retrospective analysis was approved by our institutional review board committee (approval number: 34–299), and the requirement for written informed consent was waived. Between April 2017 and October 2020 TIVAPs included non-silver-mixed venous access ports (non-silver-mixed port; NS group; Argyle Fukuroi™ Microneedle Port; Cardinal Health, Dublin, OH). NS ports were switched with S ports (silver-mixed port; S group; Argyle Fukuroi™ Microneedle Port Silver Type; Cardinal Health, Dublin, OH) in our institution in November 2020. S ports were used from December 2020 to July 2022. All TIVAP implantation procedures with the subclavian or internal jugular vein approach were included in this study.

### TIVAP Placement Procedure

All TIVAP implantation procedures were performed in an angiography suite (Artis Zee, Siemens Healthcare, Erlangen, Germany) under maximal sterile barrier precautions. After sterilization of the surgical site with alcohol or povidone-iodine and local anesthesia with 1% lidocaine, the right subclavian vein was punctured with a 22-gauge needle supplied with this product under ultrasound guidance. Once successful venous puncture was achieved, a 0.018-inch guidewire was inserted into the superior vena cava under fluoroscopic guidance. After creating a subcutaneous pocket for the port tank, the introducer and 8-French 50-cm catheter were inserted into the superior vena cava along the guidewire. After removal of the introducer and guidewire, the catheter was cut to the appropriate length and connected to the port tank, and the port tank was implanted into the subcutaneous pocket. The incision was sutured with absorbable polyfilament polyglactine 910 (Vicryl™; Ethicon GmbH, Norderstedt, Germany) for the subcutaneous fat sutures, and polyamide 6 monofilament (Ethilon ™; Ethicon Inc., Somerville, NJ) for the skin sutures. The right subclavian vein was the preferred puncture site at our institution, whereas other veins, including the left subclavian and right/left internal jugular veins, were chosen in cases of collapse of the right subclavian vein, postoperative state of the right anterior chest wall, or predilection of patients. If the right/left internal jugular veins were chosen, a subcutaneous tunnel was created for connection between the port tank and puncture site.

### Criteria of TIVAP-related Infection

Once TIVAP-related infection was clinically suspected, TIVAP was removed and cultured following blood culture. TIVAP-related infections were classified and defined according to either of the following criteria [7]:

1. Local infection: a positive port tank or catheter tunnel culture result.

2. Blood infection (bacteremia or fungemia): more than one positive blood culture with no other apparent source of bloodstream infection.

### Evaluation

Basic characteristics of the NS and S groups, including age, sex, laboratory data (albumin, absolute neutrophil count, and C-reactive protein) at the time of the procedure, use of steroids, medical history of diabetes mellitus, purpose of TIVAP implantation (chemotherapy or nutrition), primary site of cancer (colon and intestinal, head and neck, hepatobiliary pancreatic, gastric, esophageal, breast, gynecology, and lung cancers, hematological disease, urinary, primary brain tumor, soft tissue, and bone malignancies) and other benign diseases, and procedure details (length of implanted catheter and location of catheter tip) were collected. These characteristics were compared between patients with and without TIVAP-related infections to identify the risk factors for infection.

### Statistics

Statistical analyses were performed using EZR statistical software (Saitama Medical Center, Jichi Medical University, Saitama, Japan; http://www.jichi.ac.jp/saitama-sct/SaitamaHP.files/statmedEN.html) [8]. Categorical variables are presented as number of cases (percentage), and numerical variables are presented as average ± standard deviation. Comparisons were performed using Fisher’s exact test for categorical variables and Student’s t-test for numerical variables. Simple (univariate) and multiple logistic regression (multivariate) analyses were performed to identify risk factors for infection. Statistical significance was set at *p* < 0.05.

## Results

### TIVAP-related Infection Rate

The baseline demographics of the entire group are described in Table 1. The baseline demographics of the S and NS groups are summarized in Table 2. A total of 607 TIVAP placement procedures (203 and 404 procedures in the S and NS groups, respectively) were reviewed. A total of 593 procedures were carried out in an in-hospital setting. In the NS group, 31 cases (7.7%) met the criteria of TIVAP-related infection with an average time from TIVAP implantation to infection of an average of 160.2 days in an average of 359.3 days of the follow-up period. The S group demonstrated 6 cases (3.0%) of TIVAP-related infection at an average of 107.0 days after implantation in an average of 259.1 days of the follow-up period. The incidence of total infection per 1000 catheter-days were 0.114 and 0.214 in the S and NS groups, respectively. The total rates of infection were 6.1% (n = 37). The total incidence of total infection per 1000 catheter-days was 0.187. There were 0 and 5 cases of port infection within 30 days in the S and NS groups, respectively. The NS group had a significantly higher rate of TIVAP-related infection (*p* = 0.0294), lower albumin level (*p* < 0.01), higher percentage of head and neck cancer (*p* < 0.01), lower percentage of esophageal cancer (*p* < 0.0443), lower percentage of hematological disease (*p* < 0.01), lower percentage of lung cancer (*p* = 0.0149), higher percentage of other benign diseases (*p* = 0.0113), preoperative antibiotics (p = 0.0135), and shorter catheter length (*p* < 0.01).

**Table 1:** Clinical characteristics of the entire group

	Entire Group (*n* = 607)
Age over 65 years old, *n* (%)	337 (55.5)
Male Sex, *n* (%)	335 (55.2)
Steroids, *n* (%)	17 (2.8)
Diabetes Mellitus, *n* (%)	101 (16.6)
Alb (g/dl), Mean ± SD	3.4 ± 0.7
CRP (mg/dl), Mean ± SD	2.2 ± 4.2
Nutrition, *n* (%)	170 (28.0)
ANC < 1500 cells/mm^3^, *n* (%)	36 (5.9)
Underlying Disease	
Colon and Intestinal Cancer, *n* (%)	169 (27.8)
Head and Neck Cancer, *n* (%)	43 (7.1)
Hepatobiliary Pancreatic Cancer, *n* (%)	159 (26.2)
Gastric Cancer, *n* (%)	32 (5.3)
Esophageal Cancer, *n* (%)	24 (4.0)
Breast Cancer, *n* (%)	19 (3.1)
Gynecologic Cancer,* n* (%)	67 (11.0)
Hematological Disease, *n* (%)	29 (4.8)
Lung Cancer, *n* (%)	20 (3.3)
Urothelial and Kidney Cancer, *n* (%)	7 (1.2)
Primary Brain Tumor, *n* (%)	7 (1.2)
Soft Tissue and Bone Malignancies, *n* (%)	4 (0.7)
Other Benign Disease, *n* (%)	27 (4.4)
Procedure Details	
Right Sided Implantation, *n* (%)	560 (92.3)
Length of Catheter (cm), Mean ± SD	17.3 ± 3.2
Preoperative Antibiotics, *n* (%)	237 (39.0)
TIVAP-Related Infection	
Infection Rate, *n* (%)	37 (6.1)
Infection per 1000 Catheter-Days	0.187

*SD* Standard Deviation, *Alb* Albumin, *CRP* C-reactive protein, *ANC* Absolute neutrophil count, *TIVAP* Totally implantable venous access port

**Table 2:** Comparison of the clinical characteristics between the NS-group and S-group

	S-group (*n* = 203)	NS-group (*n* = 404)	*p*-value
Age over 65 years old, *n* (%)	113 (55.9)	224 (55.3)	0.931
Male Sex, *n* (%)	110 (54.4)	225 (55.8)	0.667
Steroids, *n* (%)	5 (2.5)	12 (3.0)	0.801
Diabetes Mellitus, *n* (%)	37 (18.1)	64 (15.8)	0.489
Alb (g/dl), Average ± SD	3.5 ± 0.7	3.3 ± 0.8	< 0.01*
CRP (mg/dl), Average ± SD	1.7 ± 3.7	2.4 ± 4.5	0.0577
Nutrition, *n* (%)	40 (20.1)	130 (32.3)	< 0.01*
ANC < 1500 cells/mm^3^, *n* (%)	15 (7.4)	21 (5.2)	0.28
Underlying Disease			
Colon and Intestinal Cancer, *n* (%)	65 (31.8)	104 (25.7)	0.125
Head and Neck Cancer, *n* (%)	5 (2.5)	38 (9.4)	< 0.01*
Hepatobiliary Pancreatic Cancer, *n* (%)	48 (23.6)	111 (27.4)	0.329
Gastric Cancer, *n* (%)	12 (5.9)	20 (4.9)	0.701
Esophageal Cancer, *n* (%)	13 (6.4)	11 (2.7)	0.0443*
Breast Cancer, *n* (%)	4 (2.0)	15 (3.7)	0.326
Gynecologic Cancer,* n* (%)	19 (9.3)	48 (12.1)	0.411
Hematological Disease, *n* (%)	17 (8.3)	12 (3.0)	0.01*
Lung Cancer, *n* (%)	12 (5.9)	8 (2.0)	0.0149*
Urothelial and Kidney Cancer, *n* (%)	2 (1.0)	5 (1.2)	1
Primary Brain Tumor, *n* (%)	2 (1.0)	5 (1.2)	1
Soft Tissue and Bone Malignancies, *n* (%)	1 (0.5)	3 (0.7)	1
Other Benign Disease, *n* (%)	3 (1.5)	24 (5.9)	0.0113*
Procedure Details			
Right Sided Implantation, *n* (%)	190 (93.6)	370 (92.3)	0.33
Length of Catheter (cm), Average ± SD	18.6 ± 2.7	16.6 ± 3.3	< 0.01*
Preoperative Antibiotics, *n* (%)	65 (32.0)	172 (42.6)	0.0135*
TIVAP-related Infection			
Infection Rate, *n* (%)	6 (3.0)	31 (7.7)	0.0294*
Infection per 1000 Catheter-Days	0.114	0.214	

*SD* Standard Deviation, *Alb* Albumin, *CRP* C-reactive protein, *ANC* Absolute neutrophil count, *TIVAP* Totally implantable venous access port

^*^Statistically significant

### Risk Factors of TIVAP-related infection

Intergroup univariate comparisons between the presence and absence of TIVAP-related infections showed significantly lower infection rates in the S group (Table 3). Other characteristics, including age, sex, albumin level, C-reactive protein level, steroids, diabetes mellitus, purpose of TIVAP implantation, primary site of cancer, catheter length, and location of catheter tip, did not demonstrate significant differences. Multivariate comparison with the S group (Table 4) and absolute neutrophil count < 1500 cells/mm^3^ and steroids showing a lower *p*-value in Table 3 revealed a significant association between TIVAP-related infection and the S-NS group (*p* = 0.0216, odds ratio = 2.88 confidence interval: 1.17–7.08).

**Table 3:** Comparison of the clinical characteristics between the infection group and non-infection group

	Infection (*n* = 37)	Non-infection (*n* = 570)	*p-*value
Age over 65 years old, *n* (%)	19 (51.4)	319 (55.8)	0.613
Male Sex, *n* (%)	20 (54.1)	315 (55.3)	1
Silver-mixed port, *n* (%)	6 (16.2)	197 (34.6)	0.0294*
Steroids, *n* (%)	3 (8.1)	14 (2.5)	0.0784
Diabetes Mellitus, *n* (%)	3 (8.11)	98 (17.1)	0.177
Alb (g/dl), Average ± SD	3.4 ± 0.7	3.2 ± 0.7	0.153
CRP (mg/dl), Average ± SD	2.2 ± 4.3	2.0 ± 2.5	0.729
Nutrition, *n* (%)	15 (40.5)	155 (27.2)	0.0898
ANC < 1500 cells/mm^3^, *n* (%)	5 (13.5)	31 (5.4)	0.0599
Underlying Disease			
Colon and Intestinal Cancer, *n* (%)	9 (24.3)	160 (28.1)	0.708
Head and Neck Cancer, *n* (%)	4 (10.8)	39 (6.8)	0.323
Hepatobiliary Pancreatic Cancer, *n* (%)	6 (16.2)	153 (26.8)	0.18
Gastric Cancer, *n* (%)	2 (5.4)	30 (5.3)	1
Esophageal Cancer, *n* (%)	0 (0)	24 (4.2)	0.389
Breast Cancer, *n* (%)	2 (5.4)	17 (3.0)	0.324
Gynecologic Cancer,* n* (%)	5 (13.5)	62 (10.9)	0.588
Hematological Disease, *n* (%)	1 (2.7)	28 (4.9)	1
Lung Cancer, *n* (%)	2 (5.4)	18 (3.2)	0.346
Urothelial and Kidney Cancer, *n* (%)	0 (0)	7 (1.2)	1
Primary Brain Tumor, *n* (%)	1 (2.7)	6 (1.1)	0.357
Soft Tissue and Bone Malignancies, *n* (%)	0 (0)	4 (0.7)	1
Other Benign Disease, *n* (%)	4 (10.8)	23 (4.2)	0.0746
Procedure Details			
Right Sided Implantation, *n* (%)	34 (91.9)	527 (92.7)	0.754
Length of Catheter (cm), Average ± SD	18.0 ± 3.0	17.2 ± 3.3	0.155
Preoperative Antibiotics, *n* (%)	18 (48.6)	219 (38.4)	0.227

*SD* Standard Deviation, *Alb* Albumin, *CRP* C-reactive protein, *ANC* Absolute neutrophil count

^*^Statistically significant

**Table 4:** Multivariate analysis of Silver-mixed port, ANC < 1500 cells/mm^3^ and Steroids

	Odds ratio	95% Confidence Interval	*p-*value
Silver-Mixed Port	2.88	1.17–7.08	0.0216*
ANC < 1500 cells/mm^3^	2.81	0.998–7.90	0.0505
Steroids	3.12	0.833–11.7	0.0913

*ANC* Absolute neutrophil count

^*^Statistically significant

### Microbiological Outcome

Microbiological data are shown in Table 5. Thirty-five patients had monomicrobial infection. Two patients demonstrated polymicrobial infection in the NS group: one case of *Klebsiella pneumonia* and *Candida parapsilosis* and one case of *Staphylococcus caprae* and *Enterococcus faecalis*. Gram-negative rods (GNRs) were not detected in the S group, whereas GNRs were found to be the infecting microorganisms in six patients in the NS group.

**Table 5:** Bacteriologic data of port infection between NS-group and S-group

		S-Group (*n* = 6)	NS-Group (*n* = 31)
Local Infection		6	27
Bloodstream Infection		6	24
Gram-positive Bacteria			
	*Staphylococcus aureus*	6	17
	*Enterococcus faecium*	0	2
Gram-negative Bacteria			
	*Klebsiella pneoumoniae*	0	3
	*Klebsiella oxytoca*	0	2
	*Stenotrophomonas maltophilia*	0	2
Candida		0	5

## Discussion

This single-center retrospective study demonstrated promising anti-infectious outcomes of the silver-mixed TIVAP device, as the silver-mixed TIVAP (S group) was found to be the most correlated factor for TIVAP-related infection. The TIVAP-related infection rates were 3.0%, 7.7%, and 6.1%, and the incidence of total infection per 1,000 catheter-days were 0.114, 0.214, and 0.187 in the S group, NS group, and entire group, respectively. Previous investigations reported a TIVAP-related infection rate between 5.6% and 13% and an infection rate ranging from 0.15 to 0.39/1000 catheter-days in oncological patients [9, 10]. Our study showed a lower infection rate in the S group and an equal infection rate in the NS group compared with those in previous reports. The TIVAP devices used in the S and NS groups were made of silicone with the same size and shape; the only difference was the presence or absence of silver mixed in the catheter and tank. Patients who used TIVAPs made of silicone tended to have a lower infection rate than those who used TIVAPs made of polyurethane [11]. Therefore, TIVAP made of silicone mixed with silver may be more tolerant to infection than other products.

In this study, no GNR or *Candida* infections occurred in the S group, whereas the NS group showed 7 (23%) and 5 cases (16%) of 31 TIVAP-related infections caused by GNR and *Candida*, respectively. Neutropenia due to intensified antineoplastic chemotherapy, translocation of microorganisms from the gut to bloodstream due to total parenteral nutrition (TPN), and prolonged administration of broad-spectrum antibiotics can lead TIVAP-related infection by GNR and *Candida* [12]. The antimicrobial potential of silver cations depends on the composition and thickness of the bacterial external envelope. Gram-negative bacteria may be more susceptible to the antimicrobial effect of silver because they have thinner cellular walls than gram-positive strains [13]. This difference in bacterial structure may have reduced the GNR infection rate in the S group.

Several reports are available on silver-coated or silver-impregnated medical devices, such as central venous catheters (CVCs) [14, 15], urethral catheters [5], and implants for hip arthroplasty [6]. Silver-impregnated collagen cuffs significantly decrease the risk of short-term catheter colonization [16], but fail to prevent long-term colonization, possibly because of the early degradation of the cuffs [17]. For CVCs, although a prospective randomized trial showed results supporting the anti-infectious effect of silver-impregnated catheters [15], a meta-analysis disagreed its validity [14]. In the environment of CVCs, the access site is exposed and constantly contacts gram-positive bacteria on the skin, which may have compromised the antimicrobial effect of silver-coated CVCs, because silver has more effective antimicrobial activity against gram-negative infections. Additionally, the silver-mixed TIVAP, which was used in the S group, is speculated to be quite different from silver-coated or silver-impregnated medical devices because the silver-based inorganic antimicrobial agent is kneaded into the silicone material itself. This manufacturing difference may lead to more stable and effective antimicrobial activity in vivo relative to silver-coated or silver-impregnated devices.

Previous investigations have suggested that TPN, age over 65 years, hematological disease, and cancer with an oropharyngeal and pulmonary origin are risk factors for TIVAP-related infection [7, 11, 18–20]. Additionally, immunosuppressive status, such as neutropenia and long-term steroid usage, has been reported as a factor associated with infection [20–24]. In this study, the S-NS was the only factor significantly associated with TIVAP-related infections. Multivariate analyses showed no confounding factors with this relationship. Other factors, including TPN, steroid use, age, neutropenia and primary lesions, were not statistically significant.

This study has several limitations. First, this was a retrospective, single-center study with a relatively small sample size. Second, although the observation period for the S group was above the average days of infection in the NS group, a shorter observation period in the S group may have led to an underestimation of the infection rate. Third, more detailed statistical analyses, such as addition of confounding factors, including proficiency of the operators and surgical time; adoption of exclusion criteria, including immune status and origin of malignancy; and employment of propensity matched analyses; were not feasible in this study owing to the limited sample size. Thus, a larger number of study participants is desired.

## Conclusion

TIVAPs made of silicone mixed with silver-based inorganic antimicrobial agents might reduce the risk of TIVAP-related infection, particularly GNR infection.
